# Supplementary material for: Synthesis and Characterization of Magnetic Molecularly Imprinted Polymer Sorbents (Fe3O4@MIPs) for Removal of Tetrabromobisphenol A
Source: Int J Mol Sci. 2025 Aug 8;26(16):7686. doi: 10.3390/ijms26167686 (PMC12386876; doi:10.3390/ijms26167686)
Supplement: Supplementary file 1 [file ijms-26-07686-s001.zip › ijms-3768900-supplementary.pdf]

Supplementary Materials

Synthesis and Characterization of Magnetic Molecularly Imprinted Polymer Sorbents (Fe_3O_4@MIPs) for Removal of Tetrabromobisphenol A

Clarissa Ciarlantini [1], Susanna Romano [2], Gian Marco Amici [1], Elisabetta Lacolla [1], Iolanda Francolini [1], Anna Maria Girelli [1], Andrea Martinelli [1] and Antonella Piozzi [1,*]

[1] Department of Chemistry, Sapienza University of Rome, P. le Aldo Moro 5, 00185 Rome, Italy; clarissa.ciarlantini@uniroma1.it (C.C.); marcogiam123@gmail.com (G.M.A.); elisabetta.lacolla@uniroma1.it (E.L.); iolanda.francolini@uniroma1.it (I.F.); annamaria.girelli@uniroma1.it (A.G.); andrea.martinelli@uniroma1.it (A.M.)

[2] Department of Industrial, Electronic and Mechanical Engineering Roma Tre University, Via Vito Volterra 62, Rome 00146, Italy; susanna.romano@uniroma3.it

* Correspondence: antonella.piozzi@uniroma1.it; Tel.: +39-06-4991-3692

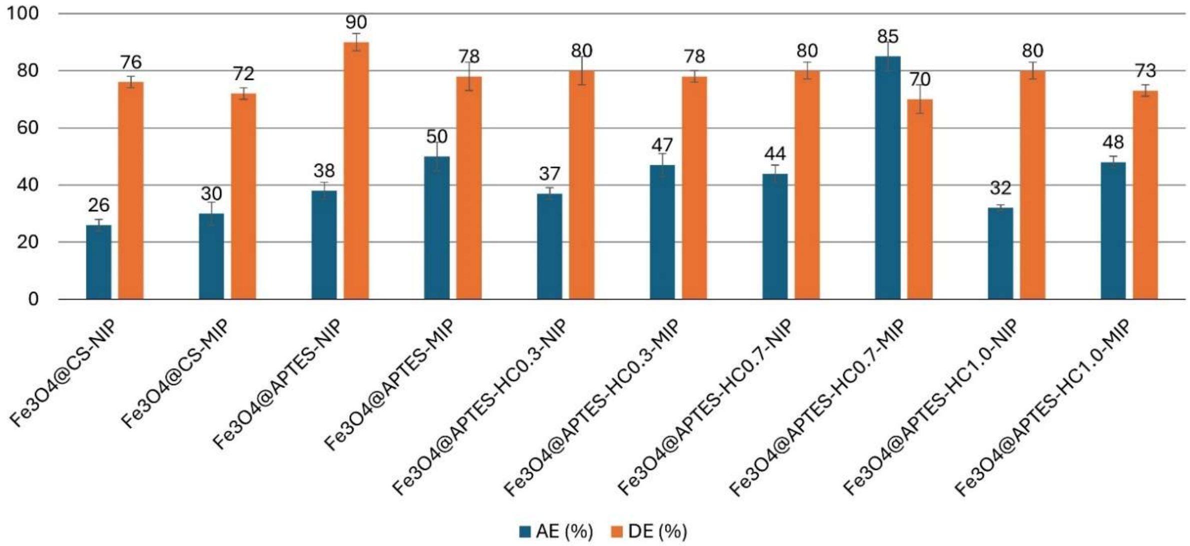

Figure S1. Adsorption efficiency AE (%) and desorption efficiency DE (%) of prepared NIP and MIP systems.

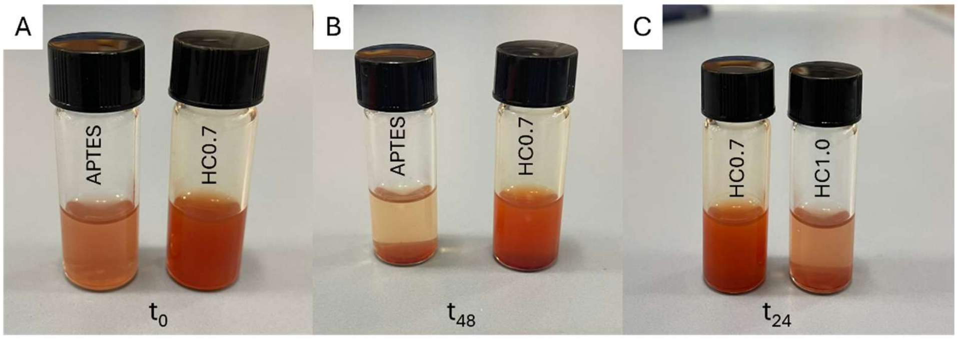

Figure S2. Images showing the dispersibility over time of the Fe_3O_4@APTES-MIP sample (left) compared to that of the Fe_3O_4@APTES-HC0.7-MIP system (right): (A) time t=0 h; (B) t=48 h. (C) Stability of the

Fe_3O_4@APTES-HC0.7-MIP sample (left) compared to that of the Fe_3O_4@APTES-HC1.0-MIP system (right) at time t=24 h.

Table S1. Affinity (α) of prepared MIP and NIP systems.

**Sample**	$\boldsymbol{\alpha}$
Fe_3O_4@CS-MIP/NIP	1.2 ± 0.1
Fe_3O_4@APTES-MIP/NIP	1.4 ± 0.2
Fe_3O_4@APTES-HC0.3-MIP/NIP	1.1 ± 0.1
Fe_3O_4@APTES-HC0.7-MIP/NIP	2.0 ± 0.3
Fe_3O_4@APTES-HC1.0-MIP/NIP	1.4 ± 0.2

Table S2. Selectivity (α) of Fe_3O_4@APTES-HC0.7-MIP/NIP system for TBBPA, 4BP and 2,4DBP.

Sample	ε (TBBPA/4BP)	ε (TBBPA/2,4DBP)
Fe_3O_4@APTES-HC0.7-NIP	1.0	0.8
Fe_3O_4@APTES-HC0.7-MIP	1.6	1.3

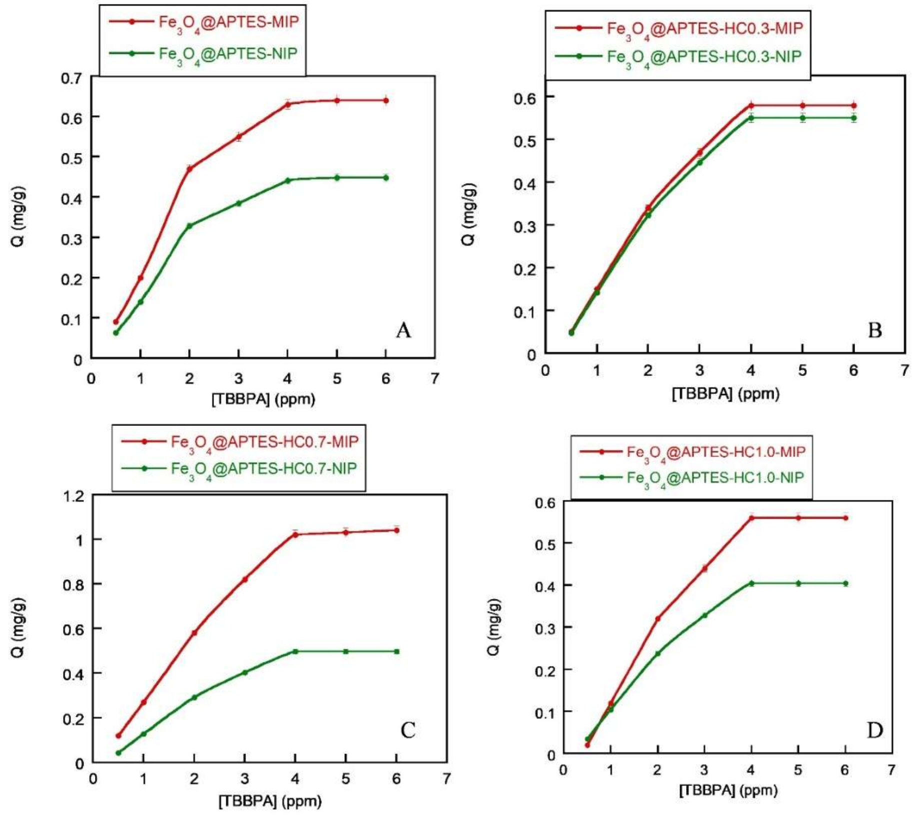

Figure S3. Comparison of adsorption isotherms of the produced MIP and NIP systems.

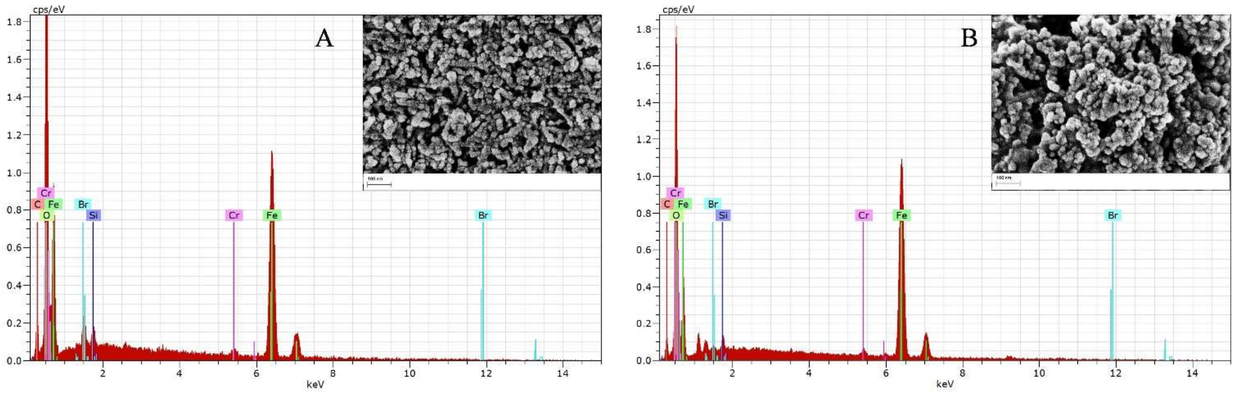

Figure S4. EDX spectra of the Fe_3O_4@APTES-HC0.7 sample after MIP formation (A) and after numerous washes with acetonitrile:H_2O (50:50).
